# Neuroendocrine carcinoma of the breast: a review of 126 cases in China

**DOI:** 10.1186/s40880-017-0211-x

**Published:** 2017-05-11

**Authors:** Yiqun Li, Feng Du, Wenjie Zhu, Binghe Xu

**Affiliations:** Department of Medical Oncology, National Cancer Centre/Cancer Hospital, Chinese Academy of Medical Sciences and Peking Union Medical College, Beijing, 100021 P. R. China

**Dear editor,**

Neuroendocrine carcinoma of the breast (NECB) accounts for approximately 0.3%–0.5% of all breast cancers [1, 2]. Due to the rarity of NECB, current understanding of this disease in China is limited to case reports and small case series, and large data analysis is still lacking. Therefore, we conducted the most comprehensive literature search to date, aiming to analyze the clinicopathologic characteristics as well as treatment and outcome of NECB in the Chinese population.

In the present study, we reported seven NECB cases diagnosed between 1990 and 2015 at the National Cancer Center & Cancer Hospital, Chinese Academy of Medical Sciences and Peking Union Medical College, Beijing, China. Additionally, we performed a literature search of the Wanfang and Weipu databases (using the following key words: breast, neuroendocrine, tumor/carcinoma/neoplasm/cancer, primary tumor/carcinoma/neoplasm/cancer, and breast tumor/carcinoma/neoplasm/cancer). Articles published between 2003 and 2015 were collected and reviewed (Table 1). NECB case reports published in non-Chinese journals were not included in this study. The diagnosis was confirmed if (a) more than 50% of the tissue specimens exhibited neuroendocrine markers, and (b) ductal carcinoma in situ was identified and/or imaging examinations and extramammary sites were excluded. Descriptive statistics were calculated for epidemiologic, clinical, and pathologic features, for treatment, and for follow-up.

**Table 1:** Summary of published articles about neuroendocrine carcinoma of the breast included in this study

Year	Author	Number of cases	Publication information
2005	Zhang et al.	5	Zhongguo Zhongliu Linchuang 2005, 32 (13)
2008	Zhong et al.	3	Zhongguo Xiandai Yiyao Zazhi 2008, 10 (7)
2008	Zhou et al.	7	Lingnan Xiandai Linchuang Waike 2008, 8 (6)
2008	Cui et al.	3	Zhongguo Shiyong Waike Zazhi 2008, 28 (7)
2009	Lv et al.	1	Guoji Waikexue Zazhi 2009, 36 (7)
2009	Ren et al.	1	Zhongguo Aizheng Zazhi 2009, 19 (5)
2009	Guan et al.	1	Zhenduan Binglixue Zazhi 2009, 16 (6)
2010	Zhang et al.	1	Zhonghua Putongwaike Zazhi 2010, 25 (12)
2010	Shi et al.	1	Anmoyukangfu Yixue 2010, 01 (7)
2010	Wang et al.	1	Zhonghua Ruxianbing Zazhi 2010, 04 (1)
2010	Zhang et al.	1	Hanshao Jibing Zazhi 2010, 17 (5)
2010	Shen et al.	1	Zhonghua Putongwaike Zazhi 2010, 25 (10)
2010	Geng et al.	3	Dalian Yikedaxue Xuebao 2010, 32 (4)
2010	Jia et al.	6	Guangdong Yixue 2010, 31 (17)
2011	Zeng et al.	3	Linchuang Yu Shiyanbinglixue Zazhi 2011, 27 (6)
2011	Kuang et al.	2	Shiyong Yixue Zazhi 2011, 27 (7)
2011	Gao et al.	16	Zhonghua Binglixue Zazhi 2011, 40 (9)
2012	Li et al.	1	Aizhengjinzhan 2012, 10 (3)
2012	Zhang et al.	4	Hainan Yixue 2012, 23 (15)
2012	Zhang et al.	32	Zhongguo Zhongliu Linchuang 2012, 39 (1)
2013	Zhang et al.	1	Zhongwai Jiankang Wenzhai 2013, 10 (4)
2013	Gu et al.	3	Bengbuyixueyuan Xuebao 2013, 38 (2)
2013	Pan et al.	8	Zhongliu 2013, 33 (2)
2014	Zhong et al.	1	Zhonghua Laonianxue Zazhi 2014, 34 (16)
2014	Hou et al.	2	ZhongliuJichu Yu Linchuang 2014, 27 (3)
2014	Yu et al.	4	Linchuang ChaoshengYixue Zazhi 2014, (8)
2015	Huang et al.	7	ZhongliuYanjiu Yu Linchuang 2015, 27 (7)

*NECB* neuroendocrine carcinoma of the breast

The main clinicopathologic features of the seven cases diagnosed at our hospital are summarized in Table 2. There were six female patients and one male patient. The median age was 49 years (range, 33–78 years). All patients presented with breast lump, and one patient developed skin ulceration. Five of seven patients had the well-differentiated subtype of disease, and two patients had poorly-differentiated disease. Five patients had early-stage disease of luminal subtype. Six patients received chemotherapy and surgery, whereas one patient received chemotherapy alone. Patients with positive estrogen receptors (ERs) received endocrine therapy. The follow-up time ranged from 31 to 59 months. Five patients were alive without tumor at the last follow-up, one died of other causes, and one was lost to follow-up.

**Table 2:** Clinicopathologic features of seven patients with neuroendocrine carcinoma of the breast

No.	Age (years)	Sex	Symptom	Histology	TNM stage	Intrinsic subtype	Treatment	Follow-up outcome
1	49	F	Breast lump	Well-differentiated	T2N0M0, IIA	ER3+PR2+HER2−	Surgery, chemotherapy, and endocrine therapy	Alive without tumor
2	78	F	Breast lump	Well-differentiated	T1N0M0, IA	ER3+PR3+HER2−	Surgery, chemotherapy, and endocrine therapy	Died of other causes
3	49	F	Breast lump	Poorly-differentiated	T2N0M0, IIA	ER3+PR+HER2−	Surgery, chemotherapy, and endocrine therapy	Alive without tumor
4	58	F	Breast lump	Well-differentiated	T2N1M0, IIB	ER2+PR2+HER2−	Surgery, chemotherapy, and endocrine therapy	Alive without tumor
5	43	F	Breast lump and skin ulceration	Well-differentiated	T3N2M0, IIIA	ER−PR−HER2−	Surgery and chemotherapy	Alive without tumor
6	60	M	Breast lump	Poorly-differentiated	T4N0M1, IV	ER−PR−HER2−	Chemotherapy	Lost to follow-up
7	33	F	Breast lump	Well-differentiated	T2N1M0, IIB	ER3+PR2+HER2−	Surgery, chemotherapy, and endocrine therapy	Alive without tumor

These patients were diagnosed and treated at the National Cancer Center/Cancer Hospital, Chinese Academy of Medical Sciences and Peking Union Medical College, China

*F* female, *M* male, *ER* estrogen receptor, *PR* progesterone receptor, *HER2* human epidermal growth factor receptor 2

Our literature search yielded 126 cases of NECB, including our own. We analyzed the clinicopathologic features of 126 NECB cases in China (Table 3). The patients’ average age was 53.2 years; there were seven male patients (5.6%) and 119 female patients (94.4%). Almost all patients (124/126, 98.4%) presented with a breast lump; six patients (4.8%) had bloody nipple discharge. No unique appearance of NECB was identified on 85 patients who underwent ultrasound examination or 66 patients who underwent mammography examination. Most patients (100/126, 79.4%) underwent mastectomy. A small percentage of patients (18/126, 14.3%) underwent breast-conserving surgery. Seven patients (5.5%) underwent breast surgery; however, the exact type of surgical procedures was not provided in the corresponding reports. One patient (0.8%) received chemotherapy alone. Overall, 55 patients received adjuvant chemotherapy; of these, 53 were initially treated with regimens for infiltrative ductal carcinoma (IDC) of the breast, including 51 receiving anthracycline- and taxane-based regimens and two receiving etoposide- and cisplatin-based regimens, and two were initially treated with the commonly used regimens for small cell carcinoma (Table 4).

**Table 3:** Clinicopathologic features of 126 patients with neuroendocrine carcinoma of the breast in China

Characteristic	No. of cases (%)
Age (years)	
<50	26 (20.6)
50–59	19 (15.1)
60–69	11 (8.7)
70–79	7 (5.6)
Data unstratified	63 (50.0)
Gender	
Female	119 (94.4)
Male	7 (5.6)
Symptom	
Breast lump	124 (98.4)
Bloody nipple discharge	6 (4.8)
Nipple erosion	1 (0.8)
Dimple sign	1 (0.8)
Skin ulceration	1 (0.8)
Diagnostic examination	
Biopsy before surgery	25 (19.8)
Breast ultrasound	85 (67.5)
Mammography	66 (52.4)
Breast MRI	1 (0.8)
Chest X-ray	39 (31.0)
Whole body check	
Abdominal ultrasound	60 (47.6)
Abdominal CT	1 (0.8)
Chest CT	1 (0.8)
Pelvic ultrasound	7 (5.6)
PET/CT	1 (0.8)
Histology	
Well-differentiated (non-small cell)	52 (41.3)
Poorly-differentiated (small cell)	26 (20.6)
NM	48 (38.1)
T category	
T1	29 (23.0)
T2	39 (31.0)
T3	24 (19.0)
T4	8 (6.3)
NM	26 (20.6)
N category	
N0	49 (38.9)
N1	12 (9.5)
N2	6 (4.8)
N3	1 (0.8)
NM	58 (46.0)
M category	
M0	122 (96.8)
M1	4 (3.2)
TNM stage	
I	30 (23.8)
II	60 (47.6)
III	14 (11.1)
IV	4 (3.2)
NM	18 (14.3)
Estrogen receptor status	
Positive	102 (81.0)
Negative	23 (18.2)
NM	1 (0.8)
Progesterone receptor status	
Positive	91 (72.2)
Negative	34 (27.0)
NM	1 (0.8)
HER2 status	
Overexpressed	19 (15.1)
Not overexpressed	104 (82.5)
NM	3 (2.4)

*MRI* magnetic resonance imaging, *CT* computed tomography, *PET* positron emission tomography, *HER* human epidermal growth factor receptor, *NM* not mentioned

**Table 4:** Treatment and follow-up of 126 patients with neuroendocrine carcinoma of the breast in China

Treatment and follow-up	No. of cases (%)
Surgery	
Mastectomy	100 (79.4)
Breast-conserving surgery	18 (14.3)
Other breast surgery	7 (5.5)
No surgery (chemotherapy alone)	1 (0.8)
Axillary lymph node dissection	104 (82.5)
Neoadjuvant therapy	
Received	4 (3.2)
None	122 (96.8)
Adjuvant therapy	
Chemotherapy	55 (43.7)
Infiltrative ductal carcinoma of the breast regimen	53 (42.1)
Small cell carcinoma regimen	2 (1.6)
Radiotherapy	22 (17.5)
Endocrine therapy	80 (63.5)
Tamoxifen	71 (56.3)
Aromatase inhibitors	4 (3.2)
Tamoxifen followed by aromatase inhibitors	1 (0.8)
Regimen not mentioned	4 (3.2)
Follow-up	
Alive without tumor	101 (80.2)
Alive with tumor	9 (7.1)
Died of disease	7 (5.6)
Died of other causes	1 (0.8)
Lost to follow-up	8 (6.3)

According to the 2012 World Health Organization (WHO) classification, 52 cases were well-differentiated, and 26 were poorly-differentiated, the remaining 48 had no information of pathology. The percentages of patients with different stage breast cancer were as follows: stage I, 23.8% (30 of 126); stage II, 47.6% (60 of 126); stage III, 11.1% (14 of 126); and stage IV, 3.2% (4 of 126); the remaining 18 had no information about pathology. ER and progesterone receptor were present in 102 (81.0%) and 91 (72.2%) patients, respectively; and human epidermal growth factor receptor 2 (HER2) protein was overexpressed in 19 (15.1%) patients.

All cases were positive for at least one of the neuroendocrine markers (chromogranin A [CgA], synaptophysin [Syn], and neuron-specific enolase [NSE]) in more than 50% of tumor cells. Table 3 summarizes the clinicopathologic features of the 126 cases.

The follow-up time ranged from 4 to 144 months. Disease recurrence was found in 13 cases, including 4 small cell type (poorly-differentiated) cases and 9 non-small cell type (well-differentiated) cases. Seven patients died of NECB, of which four had small cell type disease and three had non-small cell type disease. The treatment and follow-up of the 126 cases are shown in Table 4.

For NECB patients in China, we determined an average age of 53.2 years, which seems to be older than the onset of IDC [3]. Six patients in our study presented with bloody nipple discharge. Kawasaki et al. [4] examined the pathology of 89 patients who came to the hospital for a thorough examination of symptomatic bloody nipple discharge and found that 24 (27.0%) of them had neuroendocrine carcinomas. NECB may account for an important share of breast conditions associated with bloody nipple discharge. In previous reports, NECB showed no difference when compared with IDC based on imaging [5, 6], which was confirmed in the present study. In addition, in our study, most NECB cases showed positive ER expression, which supported the results of recent studies on gene expression profiling [7], suggesting that NECB belongs to the luminal type.

Currently, there is no standard therapy for NECB. Most treatments of NECB reported in the literature and in the present study are similar to the treatment of ductal-type carcinoma, with surgery as the first-line therapy, followed by anthracycline- and taxane-based chemotherapy and endocrine therapy [1, 8]. However, whether NECB patients can benefit from chemotherapy is unknown. Current data provide little evidence to support the use of regimens for either small cell or non-small cell carcinoma.

Conflicting results of the prognosis of patients with NECB have been reported [8–10]. Among 126 Chinese cases included in the present study, nine of 18 patients (50.0%) with the small cell carcinoma were alive without tumor relapse, whereas the percentage was 85.0% (51/60) for those with non-small cell carcinoma. Overall, 57.1% (4/7) of patients who died of NECB had small cell breast cancer.

In summary, since NECB was first listed by the WHO in 2003 as a separate unique category, many NECB cases remain to be elucidated about their etiology and treatment. In this study, we found that the onset age of NECB patients in China seems to be older than that of IDC patients. Bloody nipple discharge may indicate the existence of NECB. Most NECB patients have the luminal subtype disease. Surgery is used as the first-line therapy, and the role of chemotherapy is still unknown. The small cell subtype may be associated with more frequent relapse and a higher mortality compared with the non-small cell subtype.

## Abbreviations

NECB: neuroendocrine carcinoma of the breast
IDC: infiltrative ductal carcinoma
WHO: World Health Organization
ER: estrogen receptor
HER2: human epidermal growth factor receptor 2
CgA: chromogranin A
Syn: synaptophysin
NSE: neuron specific enolase
